# The Effect of Threshold Loading Training and an Innovative Respiratory Training Devices with Lower Torso Sports Training in Asthma Patients: A Randomized Trial

**DOI:** 10.1155/2023/3049804

**Published:** 2023-02-18

**Authors:** Shimal H. Hamad, Ammar Hamza Hadi, Magni Mohr, Suman Pandey Mahadevan, Mazin Hadi Kzar

**Affiliations:** ^1^Department of Physical Education, Soran University, 44001, Iraq; ^2^Faculty of Physical Education & Sport Sciences, University of Babylon, 11707, Iraq; ^3^Department of Sports Science and Clinical Biomechanics, SDU Sport and Health Sciences Cluster (SHSC), University of Southern Denmark, Odensea, Denmark; ^4^Centre of Health Science, Faculty of Health, University of the Faroe Islands, Tórshavn, Faroe Islands; ^5^Department of Sports and Physical Education, Savitribai Phule Pune University, Maharashtra 411007, India; ^6^Physical Education and Sport Sciences Department, Al-Mustaqbal University College, 51001, Iraq

## Abstract

This study investigated the influence of two different devices with lower torso sports training in patients with asthma. Patients with asthma (*n* = 300) aged 55-60 years with FEV1/FVC ratio < 65%, who were repeatedly admitted to a pulmonary rehabilitation centre, participated. Patients were evaluated and randomized into two groups (experimental group 1; EXP-1, *n* = 150, who applied a conventional threshold loading device, and experimental group 2; EXP-2, *n* = 150, who used an innovative respiratory training device). Patients were included only if they met the global criteria for asthma. The experimental intervention period lasted 10 weeks with 3 weekly training sessions lasting 30-40 min. The maximal inspiratory pressure (PI, max), pulmonary function test, baseline dyspnoea index (BDI), oxygen saturation, and 6 min walking test (6MWT) performance were all measured at baseline and postintervention. Also, an assessment of the 1 min repeated exercise performance (leg extension and leg press) was performed. Moreover, St. George Respiratory Questionnaire was used to quantify the quality of life (SGRQ). Statistical analysis displayed significant favourable effects on 6MWT, leg press, and FRV1, for patients using both devices (EXP-1 and EXP-2, respectively) with lower torso athletic training. The other variables, weight SPO2 and SGRO, also showed no significant change in neither EXP-1 nor EXP-2. Thus, the new respiratory training device (EXP-2) appeared to be as effective as the conventional threshold loading device (EXP-1). In conclusion, our findings demonstrated beneficial effects of combining respiratory training with athletic training in asthma patients. Additionally, the validity of a unique respiratory training device for asthma patients was confirmed.

## 1. Introduction

Asthma is one of the most prevalent chronic respiratory disorders and is estimated to affect 262 million people globally, resulting in 461,000 fatalities [1]. In Iraq, asthma has been diagnosed in 9% of adolescents and 16% of young children [2]. The disease has a large impact on public health and significantly strains the healthcare system [3]. Due to the high incidence of asthma and the associated high cost of healthcare, it is critical to develop low-cost alternatives to traditional pharmacotherapy and adjunct therapies to complement existing treatment regimens for controlling asthma and changing its severity.

Breathing exercises (BE) performed with the aid of equipment such as threshold loading training (TLT) and innovative respiratory training (IRT) and lower torso sports training (LTST) are nonpharmacological interventions that have been shown to improve asthma conditions [4]. Sport exercises, with or without equipment, have been widely employed because they are simple to implement, inexpensive, safe, and are critical adjuncts to asthma treatment [5]. Experts have routinely applied threshold loading device exercise without lower torso exercise to control asthmatic breathing symptoms. Moreover, the approach frequently appears in educational programs as self-training techniques that aim to acclimate asthma patients to an appropriate breathing pattern with a slower respiratory rate and longer expiration and inspiration, thereby decreasing hyperinflation [6]. Respiratory exercises have been proven to improve lung function, functional capacity, exercise capacities, such as the 6 min walk test, respiratory muscle strength, and health-related quality of life [7].

Threshold loading training (TLT) and novel methods of respiratory training (IRT) are quantitative techniques used to improve the strength of the inspiratory muscles during inspiration. Exercise with respiratory devices produces an effect by adjusting the inspiratory muscles to overcome resistance. Training using a threshold loading device is likely to decrease exertional dyspnoea, increase diaphragm function and structure, and decrease the oxygen cost of breathing in asthma patients [8]. In addition, threshold loading exercise has been demonstrated to upregulate respiratory muscle endurance and strength, pulmonary function, exercise capacity, functional ability, and quality of life [9].

LTST is also a critical training routine for individuals with asthma since it improves physical capacity and health-related quality of life. The guideline from the British thoracic society for the physiotherapy management of asthma patients recommends athletic training to improve cardiorespiratory endurance and fitness and overall health-related quality of life [10]. Asthma patients can benefit from physical exercise by increasing their airway reserve and decreasing their bronchospasm threshold, effectively reducing air trapping by repositioning the diaphragm [11].

Some asthma patients may show less tolerance to lower torso exercise because of worsening asthma symptoms. This may prevent them from attempting to keep fit. Upper or lower torso training programs for asthma patients have been designed to improve muscle function, muscle coordination, physical fitness, and confidence and reduce symptoms of dyspnea [12]. Lower torso training in asthma patients is essential to improve the respiratory system and muscle function and reduce dyspnea. Some studies showed that training a lower limb reflects thoracoabdominal motion and significantly decreases dyspnea and other variables [13]. There is strong evidence that leg exercise performance decreases dyspnea and leg fatigue in patients with a respiratory system. Moreover, peripheral and respiratory muscles are essential in limiting muscular performance [14]. Fiona and colleagues found that lower-limb training may help rehabilitate patients with chronic airflow obstruction and its effect on exercise performance and general well-being.

Studies on pulmonary disease patients have demonstrated that lower limb muscles were more affected than upper limb muscles. Thus, the average quadriceps strength reduction was 30% compared to healthy individuals [15]. Endurance training for the lower torso was considered a therapy modality to alleviate several symptoms experienced by patients with respiratory systems [16]. Currently, there is insufficient knowledge on possible benefits from combined inspiratory muscle training using a threshold loading device and lower torso exercise, which may be helpful for patients with asthma. Others have found that six months of inspiratory threshold loading training, added to general exercise reconditioning, markedly improved exercise tolerance, as well as inspiratory muscle endurance and strength in C.O.P.D. patients and that the improvement in this group of patients was significantly more significant than that achieved with general exercise reconditioning alone [17]. Moreover, Tounsi et al. [18] indicated that upper and lower limb endurance training associated with inspiratory muscle training significantly improved functional balance in patients with C.O.P.D. Thus, their findings suggest that inspiratory muscle training combined with endurance training is more effective in improving inspiratory muscle function and generating better balance control than endurance training alone. Finally, it has been demonstrated that lower limb exercise combined with inspiratory muscle training in the ambulatory rehabilitation program of myocardial infarction patients resulted in improved rehabilitation efficacy, significantly improving lower limb function [19].

Collectively, data on the effect of threshold loading with lower torso sport training on the test and pulmonary function tests are scarce and limited to those obtained from studies evaluating handgrip endurance and maximal inspiratory pressure (PI-max). Thus, it is interesting to investigate and compare two novel devices to ascertain their effect on both physical performance, pulmonary function ability, and quality of life. Therefore, this study applied a broad-spectrum test battery and is aimed at investigating the influence on both pulmonary function, physical ability, and quality of life of the LTST with IRT and LTST with TLT on patients with asthma and comparing TLT and IRT devices to test the validity of the innovative device. Thus, we are evaluating the effect of lower torso sports training combination with threshold loading device training on broad-scaled asthma symptoms. We hypothesize that TLT and LTST may affect physical ability, pulmonary function, and quality of life in asthma patients and that IRT with LTST may also impact these parameters.

## 2. Materials and Methods

### 2.1. Participants

Medical professionals referred three hundred thoracic outpatients presenting a follow-up clinic at a regional medical centre in Al-Hillah City/Babylon government hospital and volunteered to participate in this study (Table 1). They were aged between 55 and 60 years and had been clinically diagnosed with asthma (FEV1 ratio 65%) [20] for more than one year by a pulmonologist (see Table 1). Patients were included in the study only if they met the global criteria for asthma recommendations for asthma diagnosis, categorization, pulmonary function evaluation, and physical examination [21, 22]. Participants were included in the trial only if they were in a clinically stable state with no history of illness or worsening of respiratory symptoms, no medication alterations within the preceding two months, and no clinical signs of oedema [23]. Patients with evidence of cardiovascular, osteoarticular, or other terminal cancers, inability to consent or cooperate, long-term home oxygen therapy, active tuberculosis, or other infectious disorders, as well as stroke, were excluded. The study was approved by the Institutional Review Board (IRB) of the University of Babylon in Iraq (date of approval in April 2020; number PV3746. The study was registered as a clinical trial at the hospital and University of Babylon using the ethical registration number PV3746. All experimental procedures regarding testing, lower torso training, and respiratory training by devices were carefully explained to the participants, and written informed consent from participants who met the inclusion criteria was obtained before the beginning of the experiment. Thus, the study was conducted under the declaration of Helsinki.

### 2.2. Experimental Design and Procedure

The study was a prospective randomized trial with two groups that compared the nonpharmacological interventions TLT and IRT with LTST. G^∗^Power 3 with a priori power analysis was performed, using a predefined power of 0.8, an effect size of 0.8, and an alpha level of 0.05 (GBD 2015). 300 participants were recruited and randomly divided into two intervention groups (experimental group 1 (EXP-1), who used TLT with LTST and experimental group 2 (EXP-2), who used IRT with LTST) with each group involving 150 asthma patients. After baseline screening and testing, all 300 eligible participants were assigned to the TLT with LTST or IRT with LTST group by a third person in a blinded manner randomly (i.e., only using the subject number without knowledge of any other identification or baseline test result) using block randomization targeting group sizes of *n* = 150. Our study is the high sample size and high adherence to the training. Block randomization works by randomizing participants within blocks such that an equal number are assigned to each treatment.

Pre- and postintervention assessments were all conducted by an expert with special respiratory training. During the 10-week program, the two groups participated in 30 to 40 min sessions three times in a week. The sessions emphasized breathing rhythm regulation, increasing LTST, increasing expiratory time, boosting diaphragmatic and nasal breathing, and slowing the respiratory flow. Each session was initiated with activities to warm up the lower torso and thoracic wall muscles. LTST was performed with TLT and IRT and with diaphragmatic and pursed-lip breathing. By utilizing the TLT (Power breathe, HaB International, Southam, UK), the patients were instructed to breathe through their mouths and clip their noses shut, and they were encouraged to exhale “the whole air content of their lungs.” Each breath was 40–60% of maximal inspiratory pressure (PImax), and LTST was 40%–60% of PImax. The patients were encouraged to maintain a modest breathing frequency to minimize hyperventilation and to gradually increase the resistive load until 40 breaths approximated the inspiratory muscle tolerance limit. To ensure that patients adhered to the training requirements of their training interventions, they were required to visit the laboratory monthly to complete their tests.

The differences between TLT and LTST machine are as follows:

1. There is a mask that covers both mouth and nose. Inhalation and exhalation are taken inside the mask without exposure to the air pollutants of the external environment. In contrast, threshold loading device is placed in the mouth only
2. Two side filters filter the air from dust and other pollutants entering the lungs. While threshold loading device does not have a filter
3. There is a pulley for timing the intensity from zero to 100%, which is suitable for all degrees of shortness of breath, while the intensity of the threshold loading device is 40% which is unsuitable for all degrees of dyspnea
4. The innovative device works with electricity or batteries, and this aids the patient in the process of inhaling air more efficiently. However, unfortunately, we do not find that in the threshold loading device
5. The innovative device can be carried back without affecting the patient's movement. However, simultaneously, the threshold loading device must be held by a hand, which may affect patient movement
6. The innovative device can be linked to a computer to read the percentage of inhaled oxygen and exhaled carbon dioxide. We do not find that in the threshold loading device

The innovative device has two gates, one for air entry and the other for air exit, while the threshold loading device has one gate.

### 2.3. Testing Procedures

The 6 min walk test and the pulmonary function test are simple objectives for determining a patient's capacity to walk as far as feasible while breathing easily. They are also an inexpensive and reliable device for estimating functional activity and submaximal exercise proficiency [24]. The 6 min walk test can be performed by senior individuals and assesses people with severe diseases such as asthma should be included in the assessment of asthma patients. Numerous factors can affect asthma patients' 6 min walk test performance, such as age, body composition, mental health, and comorbidities. Previously, it was established that muscle endurance in the lower limbs plays a significant role in 6 min walk test performance [25].

### 2.4. Pulmonary Function

The used devices: FEV1 will be used to assess lung function. A spirometer (as used by Burt et al., [26]) will be used to measure forced expiratory volume in 1 second (FEV1). The spirometer is a simple test and an essential tool in diagnosing airway obstruction. However, the variability of spirometry measurements is greater than in most other clinical laboratory tests because the result is highly dependent on the consistency of the efforts made by patients and technicians [27]. In addition, each subject will be tested according to the American Thoracic Society's criteria, which recommends using FEV1 to diagnose the severity of airway obstruction to detect lung function [28].

Test method: FEV1 was evaluated by allowing the patients to sit on a chair at the height of (40 cm), where the feet are touching the ground, and the back is straight and resting on the back of the chair. The researcher begins to teach the patient the technique of the test by taking a deep inhale and then forcefully and quickly expelling the air from the chest (exhale) until the lungs empty or until the device makes a sound, and the nose is closed with an airtight stopper to ensure that the inhalation is taken out from the mouth only. Scoring method: the patients must continue to completely expel the exhaled air until apnoea and the inability to continue, and it must be at least (6) seconds and may last up to 12 seconds or more, and the test was performed on three attempts for each patient with one-minute rest among tests recording the best performance, noting that the reading of the attempt appears directly on the device's screen, and then, it is printed to become a graph paper, and the unit of measurement is the percentage (%).

Spirometer (German type) measurements were conducted under American Thoracic Society (ATS) guidelines [29] using previously obtained reference values from healthy members of our group [30]. At each assessment, each contributor was classified as a never-smoker. In addition, the following were excluded from the 6 min walk test: regular use of an ambulatory aid (walker); inability to walk due to musculoskeletal problems; chest pain in the preceding two weeks; a heart attack or heart surgery in the preceding four months; heart rate 55 beats/min at rest (unless a physician or nurse determined that an AV block or conduction problem was not the cause of the bradycardia); heart rate 60 beats/min at rest (unless a physician, these exclusions were probably prudent (they excluded a large number of individuals who would have willingly and safely undertaken the test), as physicians could not be present in the clinics throughout all examinations to evaluate and treat symptomatic patients.

### 2.5. Nutritional Evaluation

Height and weight were determined and the body mass index (BMI) [weight/height squared]. Resistance was determined on the right side of the body in the flat posture using the [31]. Kyle and co-authors established a group-specific regression equation for calculating lean body mass (LBM) in kilograms [32].

### 2.6. Quality of Life and Baseline Dyspnoea

A validated version of the St. George Respiratory Questionnaire (SGRQ) was used to assess patient quality of life (QoL) [33] In addition, a similarly adjusted version of the (Mahler et al., 1984) baseline dyspnea index (BDI) was utilized to assess baseline dyspnea.

### 2.7. Maximum Inspiratory Pressure (PImax)

The maximum inspiratory pressure was determined as previously described [34]. At the mouth, the PImax was determined using residual volume and total lung capacity, respectively. A tube-type mouthpiece was linked to the pressure transducer (P23 ID, Gould Instrument Systems, Valley View, Ohio) through 60 cm of pressure tubing, and an air leak was created at the mouthpiece via a small hole (diameter = 1.6 mm) to reduce the contribution of buccal muscles during the manoeuvre. All manoeuvres were carried out while seated in a chair. To avoid tiredness, data collection was paced: five PImax exercises were conducted with one-minute rest intervals; patients rested for five minutes before doing an additional five PImax manoeuvres with one-minute rest intervals. The PImax trials were classified as those with the highest sustained negative and positive pressures, respectively, against an obstructed airway for 1 second. The PImax per cent predicted was determined using Black and Hyatt's prediction equations: PImax per cent predicted = 120 (0.25 age) for men.

### 2.8. Leg Extension and Leg Press Tests

The following activities were performed: leg extension (quadriceps) and leg press (quadriceps, gluteus, hamstrings, and calf muscles). Before the test, 12 repetitions with a modest weight were performed to mitigate learning effects. All participants completed the 1 min time limit on two attempts. Between repetitions, 1–2 minutes of relaxation were allowed. The Valsalva manoeuvre was avoided, and the proper technique for doing each muscle group's workout was emphasized [35].

### 2.9. The 6 Min Walk Test

According to American Thoracic Society guidelines [36], patients were instructed to walk for 6 min, covering as much ground as possible. A study assistant timed the walk and provided standardized verbal encouragement to each patient. Data were collected on Spo2, heart rate, respiratory rate, Borg scale dyspnea score, and blood pressure before and after the test. The performance in the 60 min walk test was measured in m.

### 2.10. Statistical Analysis

To investigate intervention induced between and within-group changes, ANCOVA test from the SPSS software was used. BMI was chosen as body composition measures, whilst FEV1 and Spo2 were chosen as pulmonary function metrics. As a dependent variable, the 6MWT was defined. Data are except correlation coefficients presented as means ± SD.

## 3. Results

Table 2 and 3 presents the score of ANCOVA analysis of data.

### 3.1. Body Weight

- Prior to the implementation of experimental programs, the mean of the body weight of EXP-1 and EXP-2 was 71.50 kg (±1.13) and 71.48 kg (±1.06), respectively. In the postimplementation of experiments, the mean of EXP-1 and EXP-2 was 71.14 kg (±1.22) and 71.16 (±1.16), respectively. Further, the posttest scores of both the experimental groups were compared using ANCOVA where pretest scores were covariate. Based on the analysis, comparison between the groups shows that *F* value in case of body weight is 0.126 which is not significant at 0.05 level of significance (*P* = 0.722); hence, it does not show significant difference between the EXP-1 and EXP-2 (Tables 2–4).

### 3.2. Respiratory Parameters

- The mean of FEV1 before the implementation of experiments EXP-1 and EXP-2 was 66.37 (±1.12) and 66.36 (±1.11), respectively. In the postimplementation of experiments, the mean of exp1 and exp 2 was 76.27 (±1.09) and 76.01 (±1.19), respectively. Further, the posttest scores of both the experimental groups were compared using ANCOVA where pretest scores were covariate. Based on the analysis, comparison between the groups shows that *F* value in case of FEV1 is 3.892 which is significant at 0.05 level of significance (*P* = 0.049) it shows significance difference between the effect of EXP 1 and EXP2 on FEV1
- The mean of SPO2 before the EXP-1 and EXP-2 was 90.96 (±1.21) and 91.02 (±1.13), respectively. In the postimplementation of experiments, the mean of exp1 and exp 2 was 91.56 (±0.73) and 91.50 (±0.75), respectively. Further, the pos test scores of both the experimental groups were compared using ANCOVA where pretest scores were covariate. Based on the analysis, comparison between the groups shows that *F* value in case of SPO2 is 0.644 which is not significant at 0.05 level of significance (*P* = 0.423); hence, it does not show significant difference between the EXP-1 and EXP-2 Tables 2–4)
- The mean of PImax before the implementation of experiments EXP-1 and EXP-2 was 66.30 (±1.02) and 66.36 (±1.11), respectively. In the postimplementation of experiments, the mean of EXP1 and EXP 2 was 62.65 (±1.09) and 62.89 (±1.10), respectively. Further, the posttest scores of both the experimental groups were compared using ANCOVA where pretest scores were covariate. Based on the analysis, comparison between the groups shows that *F* value in case of PImax is 3.551 which is significant at 0.05 level of significance (*P* = 0.06); it shows significant difference between the scores of EXP-1 and EXP-2 Tables 2–4)
- The mean of BDI before the implementation of EXP-1 and EXP-2 was 21.84 (±1.31) and 21.85 (±1.18), respectively. In the postimplementation of experiments, the mean of exp1 and exp 2 was 22.09 (±1.23) and 21.90 (±1.19), respectively. Further, the posttest scores of both the experimental groups were compared using ANCOVA where pretest scores were covariate. Based on the analysis, comparison between the groups shows that *F* value in case of BDI is 6.694 which is significant at 0.05 level of significance (*P* < 0.01) it shows significant difference between the scores of EXP-1 and EXP-2 (Tables 2–4)

### 3.3. Physical Performance

- Prior to the implementation of experimental programs, the mean of the leg extension of EXP-1 and EXP-2 was 19.18 (±0.83) and 19.25 (±0.83), respectively. In the postimplementation of experiments, the mean of exp1 and exp 2 was 21.43 (±1.16) and 21.24 (±1.08), respectively. Further, the posttest scores of both the experimental groups were compared using ANCOVA where pretest scores were covariate. Based on the analysis, comparison between the groups shows that *F* value in case of leg extension is 2.076 which is not significant at 0.05 level of significance (*P* = 0.151); hence, it does not show significant difference between the EXP-1 and EXP-2 (Tables 2–4)
- The mean of leg press before the implementation of experiments EXP-1 and EXP-2 was 21.82 (±1.19) and 21.90 (±1.17), respectively. In the postimplementation of experiments, the mean of EXP-1 and EXP-2 was 25.04 (±0.88) and 24.82 (±0.87), respectively. Further, the posttest scores of both the experimental groups were compared using ANCOVA where pretest scores were covariate. Based on the analysis, comparison between the groups shows that *F* value in case of leg press is 4.731 which is significant at 0.05 level of significance (*P* = 0.030); it shows significant difference between the scores of EXP-1 and EXP-2 (Tables 2–4)
- The mean of 6MWT before the implementation of experiments EXP-1 and EXP-2 was 417.30 (±25.51) and 417.50 (±25.49), respectively. In the postimplementation of experiments, the mean of EXP-1 and EXP-2 was 521.27 (±6.52) and 518.25 (±7.57), respectively. Further, the posttest scores of both the experimental groups were compared using ANCOVA where pretest scores were covariate. Based on the analysis, comparison between the groups shows that *F* value in case of 6MWT is 13.645 which is significant at 0.05 level of significance (*P* < 0.001); it shows significant difference between the scores of EXP-1 and EXP-2 (Tables 2–4)

### 3.4. Quality of Life (QOL)

Prior to the implementation of experimental programs, the mean of the SGRQ (QOL) of EXP-1 and EXP-2 was 47.37(±1.33) and 47.36 (±1.22), respectively. In the postimplementation of experiments, the mean of EXP-1 and EXP-2 was 50.42 (±1.05) and 50.23 (±1.04), respectively. Further, the posttest scores of both the experimental groups were compared using ANCOVA where pretest scores were covariate. Based on the analysis, comparison between the groups shows that *F* value in case of SGRQ (QOL) is 2.370 which is not significant at 0.05 level of significance (*P* = 0.125); hence, it does not show significant difference between the EXP-1 and EXP-2 (Tables 2–4).

From the above-mentioned findings, this can be concluded that EXP-1 and EXP-2 both had positive effect on the body weight, quality of life SPO2, PImax, and leg extension, although the difference between postexperiment scores between the groups were not significant statistically; hence, it can be said that both the experiments had similar effects on the patients which indicate that EXP-2 (respiratory training with athletic training) appeared to be as effective as the EXP-1 (conventional threshold loading device). However, for FEV1, BDI, leg press, and 6MWT, EXP-1 and EXP-2 both had positive effect, and the difference between postexperiment scores between the groups was found statistically significant with EXP-2 (respiratory training with athletic training) having better effect then EXP-1 (conventional threshold loading device).

Hence, it can be concluded that combining respiratory training with athletic training will be more beneficial for asthma patients compared to conventional treatment method.

## 4. Discussion

The results of our study demonstrated for the first time that threshold loading training and LTT are predictors of physical ability in asthma patients. Moreover, our results confirm the effect of IRT with LTST on FEV1, maximum inspiratory pressure (PImax), the quality of-life, baseline dyspnoea, and body weight on the physical capacity of this patient group.

The effects of TLT and IRT with LTST on 6MWT are well described in the literature in terms of PImax. The findings in the present study corroborate prior research with asthma patients [37, 38], demonstrating that PImax affects 6MWT. Wijkstra et al. [37] also estimated the effect of PImax, QoL, pulmonary function, and dyspnea sensation on the exercise capacity of 40 individuals with asthma and showed that PImax and diffusing capacity explained 54% of the variance in the 6MWT [39]. Although PImax reflects the pressure generated by the inspiratory muscles, its measurement is influenced by other factors such as the respiratory system's passive elastic recoil pressure, including the lungs and chest wall [36] Moreover, estimating accessory respiratory muscle endurance using an unaffected protocol by lung capacity or elastic recoil may provide additional information on these muscles' effects on the 6MWT.

The present study is the first to describe the effect of TLT and IRT with LTST on 6MWT performance. We observed a marked improvement in both groups. Others have assessed the impact of thoracic and upper limb muscular endurance on patients with respiratory illness maximal exercise capacity but did not use the 6MWT. Nonetheless, a study has described the effect of lower-limb peripheral muscle function on exercise capacity in patients with respiratory disease, including [37] the impact of lower muscle training on 6MWT may be explained because the pull-down exercise involves a significant number of accessory respiratory muscles. Indeed, the rhomboids, trapezius, latissimus dorsi, pectoralis major, and biceps brachii are all-important muscles for this exercise [38]. In addition, when the primary respiratory muscles are defective or incapable of meeting ventilator requirements, some muscle groups may perform an accessory respiratory function [39].

Our findings are consistent with previous research [40], demonstrating a significant favourable effect of lower muscle training on 6MWT performance in patients with respiratory disease and establishing this specific muscular training as a predictor of 6MWT score in healthy persons. Furthermore, since lower limb endurance and strength are direct indicators of skeletal muscular endurance and strength in the leg and distal lower-torso muscles [41, 42], our data corroborate the assertion that TLT and IRT with LTST affect walking distance and have important clinical and functional importance for this patient group.

Another indication that asthmatic systemic symptoms impair exercise ability was finding bodyweight as a predictor of 6MWT in our study. We demonstrated a statistically significant beneficial influence of body weight on the 6MWT performance, consistent with other studies [43]. According to Enright et al. [44], obese and overweight patients walked shorter distances than eutrophic individuals. Low body weight is frequently associated with decreased BMI and increased muscle endurance and strength. Additionally, obesity increases the amount of energy expended during a given workout session. As a result, any scenario may result in a diminished ability to walk greater distances. As previously shown, the baseline sensation of dyspnea, as measured by the BDI, was also a predictor of 6MWT in our patients [45]. The authors discovered that the most relevant predictors of 6MWT performance were scores on three different dyspnea instruments and the dyspnea domain of the Chronic Respiratory Disease Questionnaire. In asthma patients, Nishimura et al. [46] demonstrated that the sensation of dyspnea was a predictor of 6MWT performance, maximum oxygen uptake, and endurance capacity, which supports our findings.

It has been shown that TLT and IRT with LTST had a statistically significant effect on 6MWT score and QoL, as measured by the SGRQ activity domain total score. For example, have some trials evaluated pulmonary function, 6MWT performance, respiratory muscle endurance, body composition, and peripheral muscular endurance, and 6MWT was identified as a predictor of SGRQ activity and influence domains [47]. Also, Goldstein et al. [48] discovered a statistically significant association between QoL markers and exercise ability in individuals with asthma. These findings imply that exercise capacity is an important predictor of quality of life in individuals with asthma. Lower-limb muscle endurance influences asthma patients' submaximal and maximal exercise capacity [40]. No significant difference between the two groups was found, which indicates that the new device IRT can be deemed reliable and reasonable to use with patients with asthma.

Further, the overall effect of threshold loading in the present study is consistent with the recommendations of previous research [48] in which a special device was administered to give threshold loading to healthy subjects and asthma patients. The results of this study indicated that threshold loading had a significant effect on both groups. The authors stated in their study that threshold loading training is effective in normal individuals. Other techniques, such as resistive loading and isocapnic hyperpnea, have improved respiratory muscle function among healthy individuals and patients with lung disease. Our study is a high sample size trial with high training adherence, which is a major strength. Also, the broad-ranging measurement applied in the study is a strength. However, the study has some limitations in the age range of the sample, as well as the existence of various comorbidities, which may have impacted the training responses. Several studies have been conducted to see the effect of training on patients with respiratory problems, but in the present study, the combination of TLT with LTST and IRT with LTST was tried for the first time. These nonpharmacological methods have shown better effectiveness than the conventional methods. Although the experiments were administered for 10 weeks, long-term studies are required to evaluate the consistency and consolidation of the effects. Also, to provide support to the findings of this research, relevant clinical tests may be conducted to support the findings in the present study.

Future studies will be required to estimate the impact of IRT with upper torso training, including specific muscle reconditioning on submaximal exercise capacity in respiratory disease patients. The most effective component of pulmonary rehabilitation is associated with physical conditioning. Therefore, LTST with free weights is possible for muscle overhauling in asthma patients.

## 5. Conclusion

The effect of TLT and IRT with LTST observed in our study does essentially suggest a significant effect. Both devices with LTST are beneficial to improve inspiratory muscles and exercise capacity in asthma patients.

Our findings highlight the importance of inspiratory training and skeletal muscle in exercise capacity in asthma patients. Peripheral muscle endurance, body weight, the sensation of dyspnoea, and inspiratory muscle training all influence the capacity of asthma patients to carry out exercises and physical works. Therefore, the development of treatment strategies that, while considering individual objectives and requirements, aims to interrupt the dyspnoea, dyspnea cycle, and sedentary lifestyle in these patients is warranted.

## Figures and Tables

**Table 1:** The baseline characteristics of the entire study population (*n* = 300).

Variables	Units	Mean	SD
Age	Years	58.2	7.4
Weight	Kg	72.4	9.1
BMI	Kg/m^2^	23.1	2.7
FEV1	% of predicted	65.0	6.1
Spo2	%	90.0	8.5
BDI	Score	22.0	3.7
Total SGRQ score	%	48.6	6.4
PImax	cm H2O	66.0	8.0
Leg extension	Kg	19.0	2.8
Leg press	Kg	22.1	3.3
6MWT	m	520	82

**Table 2:** Significant differences of pre and posttests for EXP-1 and 2.

	Variables	Type III sum of squares	Df	Mean square	*F*	Sig.	Partial eta squared
1	Weight	.070	1	.070	.126	.722	<.001
2	FEV1	5.039	1	5.039	3.892	.049	.013
3	Spo2	.342	1	.342	.644	.423	.002
4	BDI	2.926	1	2.926	6.694	.010	.022
5	Total SGRQ score_	2.621	1	2.621	2.370	.125	.008
6	PImax	4.331	1	4.331	3.551	.060	.012
7	Leg extension	2.616	1	2.616	2.076	.151	.007
8	Leg press	3.665	1	3.665	4.731	.030	.016
9	6MWT_	682.673	1	682.673	13.645	<.001	.044

1. *R* squared = .617 (adjusted *R* squared = .614), 2. *R* squared = .026 (adjusted *R* squared = .019), 3. *R* squared = .051 (adjusted *R* squared = .045), 4. *R* squared = .706 (adjusted *R* squared = .704), 5. *R* squared = .010 (adjusted *R* squared = .004), 6. *R* squared = .012 (adjusted *R* squared = .005), 7. *R* squared = .013 (adjusted *R* squared = .006), 8. *R* squared = .016 (adjusted *R* squared = .009), 9. *R* squared = .047 (adjusted *R* squared = .040).

**Table 3:** Dependent mean pairwise comparisons posttest between both groups posttest.

Variables	Groups		Mean difference (*I*-*J*)	Std. error	Sig.^b^
Weight	EXP-1	EXP-2	-.030	.086	.722
FEV1	EXP-1	EXP-2	.259^∗^	.131	.049
Spo2	EXP-1	EXP-2	.068	.084	.423
BDI	EXP-1	EXP-2	.198^∗^	.076	.010
Total SGRQ score	EXP-1	EXP-2	.187	.121	.125
PImax_ post	EXP-1	EXP-2	-.240	.128	.060
Leg extension	EXP-1	EXP-2	.187	.130	.151
Leg press	EXP-1	EXP-2	.221^∗^	.102	.030
6MWT	EXP-1	EXP-2	3.017^∗^	.817	<.001

Based on estimated marginal means. ^∗^The mean difference is significant at the .05 level. b. Adjustment for multiple comparisons: Bonferroni. 95% confidence interval for difference^b^.

**Table 4:** Descriptive statistics of pre and posttest for experimental 1 and experimental 2 descriptive statistic (*n* = 300).

Group		Weight	FEV1	Spo2	BDI	Total SGRQ score	PImax	Leg extension	Leg press	6MWT
EXP-1 pretest	Mean	71.5000	66.3733	90.9667	21.8400	47.3733	66.3067	19.1800	21.8267	417.3067
	Std. error of mean	.09256	.09149	.09895	.10706	.10891	.08405	.06826	.09774	2.08349
	Std. deviation	1.13368	1.12056	1.21189	1.31118	1.33385	1.02941	.83602	1.19701	25.51745

EXP-1 posttest	Mean	76.2733	91.5667	22.0933	50.4200	62.6533	21.4333	21.4333	25.0467	76.2733
	Std. error of mean	.08919	.06014	.10071	.08632	.08974	.09479	.09479	.07225	.08919
	Std. deviation	1.09236	.73655	1.23346	1.05722	1.09903	1.16098	1.16098	.88490	1.09236

EXP-2 pretest	Mean	71.4800	66.3667	91.0200	21.8533	47.3667	66.3600	19.2533	21.9000	417.5067
	Std. error of mean	.08655	.09143	.09243	.09711	.09985	.08262	.08365	.09589	2.08188
	Std. deviation	1.06007	1.11978	1.13202	1.18936	1.22292	1.01188	1.02444	1.17439	25.49766

EXP-2 posttest	Mean	71.1600	76.0133	91.5067	21.9067	50.2333	62.8933	21.2400	24.8267	518.2533
	Std. error of mean	.09511	.09739	.06185	.09755	.08536	.09031	.08858	.07126	.61888
	Std. deviation	1.16481	1.19274	.75748	1.19477	1.04539	1.10609	1.08486	.87275	7.57972

## Data Availability

Readers can get access to the data supporting the conclusions of the study by contacting the corresponding author.

## Abbreviations

ATS: American Thoracic Society
BE: Breathing exercise
BDI: Baseline dyspnoea index
LTST: Lower torso sports training
LTE: Exercises for the lower torso
IRT: Innovative respiratory training
IRTD: Innovative respiratory training device
TLT: Threshold loading training
6MWT: 6 min walk test
PImax: Maximal inspiratory mouth pressures
FEV1: Force expiratory ventilation at the first second
Spo2: Peripheral oxygen saturation.
